# Kawasaki syndrome: an intriguing disease with numerous unsolved dilemmas

**DOI:** 10.1186/1546-0096-9-17

**Published:** 2011-07-20

**Authors:** Fernanda Falcini, Serena Capannini, Donato Rigante

**Affiliations:** 1 Department of BioMedicine, Section of Rheumatology, Transition Clinic, University of Florence, Florence, Italy; 2 Department of Pediatric Sciences, Università Cattolica Sacro Cuore, Rome, Italy

## Abstract

More than 40 years have passed since Kawasaki syndrome (KS) was first described. Yet KS still remains an enigmatic illness which damages the coronary arteries in a quarter of untreated patients and is the most common cause of childhood-acquired heart disease in developed countries. Many gaps exist in our knowledge of the etiology and pathogenesis of KS, making improvements in therapy difficult. In addition, many KS features and issues still demand further efforts to achieve a much better understanding of the disease. Some of these problem areas include coronary artery injuries in children not fulfilling the classic diagnostic criteria, genetic predisposition to KS, unpredictable ineffectiveness of current therapy in some cases, vascular dysfunction in patients not showing echocardiographic evidence of coronary artery abnormalities in the acute phase of KS, and risk of potential premature atherosclerosis. Also, the lack of specific laboratory tests for early identification of the atypical and incomplete cases, especially in infants, is one of the main obstacles to beginning treatment early and thereby decreasing the incidence of cardiovascular involvement. Transthoracic echocardiography remains the gold-standard for evaluation of coronary arteries in the acute phase and follow-up. In KS patients with severe vascular complications, more costly and potentially invasive investigations such as coronary CT angiography and MRI may be necessary. As children with KS with or without heart involvement become adolescents and adults, the recognition and treatment of the potential long term sequelae become crucial, requiring that rheumatologists, infectious disease specialists, and cardiologists cooperate to develop specific guidelines for a proper evaluation and management of these patients. More education is needed for physicians and other professionals about how to recognize the long-term impact of systemic problems related to KS.

## Background

Kawasaki syndrome (KS, OMIM 611775) is an acute necrotizing vasculitis of the medium and small-sized vessels, predominantly occurring in children aged 6 months-5 years, with a male-to-female ratio of 1.5-1.8 to 1 and a life-threatening predisposition to involve coronary arteries [1]. Though damage of coronary arteries is the main complication of the disease, systemic inflammation in many organs including myocardium, liver, lungs or kidney has been documented [2]. KS is the most severe vasculitic syndrome of childhood, typically striking children younger than age 5, but even observed in neonates, adolescents and adults [3-5], and nowadays represents the leading cause of acquired heart disease in children living in the developed countries, where it has surpassed rheumatic fever [6]. The peak age in Japan, where the disease is most common, is 5 to 11 months, while in the United States it is between 1 and 2 years of age [7]. As specific laboratory tests are unavailable, the diagnosis of KS still relies on the clinical criteria described by Kawasaki himself and listed in Table 1: fever lasting at least 5 days and four of the five typical features are required to meet the clinical diagnosis [8]. Mucous-membrane changes are the most frequent manifestations of KS, occurring in more than 90% of patients, whilst the least frequently observed criterion is cervical lymphadenopathy. In very young children diagnosis is still a challenge for general practitioners, since the disease's course is often incomplete and more difficult to recognize, with subsequent delay of appropriate therapy and possible increased risk of coronary artery aneurysms (CAA) [9].

**Table 1:** Clinical findings useful for the diagnosis of classic Kawasaki syndrome: patients with fever lasting more than five days and refractory to antibiotics must have 4 of the following 5 signs

Polymorphous erythematous rash

Bilateral conjunctival injection without exudate

Changes in oral cavity and lips, including diffuse oropharyngeal hyperemia, strawberry tongue, lip swelling, fissuring, erythema or bleeding

Nonpurulent cervical lymphadenopathy (more than 15 mm in diameter, usually unilateral)

### What the epidemiologic data show

Although reported all over the world, KS is most highly expressed among Asian populations, mainly of Japanese and Korean descent. In Japan, the 20th nationwide survey documented an annual incidence of 216.9 per 100,000 children aged 0-4 years. This incidence is remarkably greater than in Europe (2.9 to 6.9 cases per 100,000) or in the Caucasian population of the United States of America (6 to 9 cases per 100,000), supporting the speculation that both genetic predisposition and environmental factors are critical for KS pathogenesis [10]. As emerged from different surveys over different countries, the disease affects mainly children under 5 years, with a peak incidence in children 1 to 2 years of age. In addition, siblings of patients with KS have a significantly greater chance of acquiring KS than children in the general population. In Japan siblings of an index case have a 10-fold increased relative risk of KS. These familial cases may imply a risk for increased severity or recurrence of the KS, which varies according to ethnicities, being about 3% in Japan and 0.8% in the States [11,12]. A host of data show that cardiac sequelae occur more frequently after a recurrent disease than in the first episode. Furthermore, patients with a history of parental KS are prone to develop a more aggressive course, requiring additional treatment with intravenous immunoglobulins (IVIG), and have a higher incidence of cardiac complications [13].

### The etiopathogenetic studies in progress

Though many etiological agents have been proposed, the exact cause of KS continues to be elusive. The rarity of the illness in the first months of life suggests a passive protection by maternal antibodies. Seasonality of cases, nationwide epidemics, and the self limited nature of KS favor an infectious trigger. Although the subject is still much debated, current research appears to indicate that the primum movens of KS might be an infectious agent with superantigenic activity, leading to a massive stimulation of the immune system and to the development of KS in a small subset of genetically predisposed individuals [14]. The discovery of viral-like cytoplasmic inclusion bodies in ciliated bronchial epithelial cells supports the hypothesis that KS might arise from a previously unidentified ubiquitous RNA virus giving rise to a state of persistent viral infection [15].

The immune response in KS encompasses both aspects of innate and adaptive immunity with a significant overproduction of different cytokines and activation of endothelial cells. Activation of both B and T cells has been detected together with increased proinflammatory cytokine production, including tumour necrosis factor (TNF)-α, interleukin-1 and interleukin-6 [16,17]. The exact mechanisms leading from immune activation to localized coronary artery damage are still not elucidated, but considerable evidence has accumulated in the last decade to suggest that T cells play a significant role. Interferon-gamma, a pleiotropic cytokine predominantly produced by T and NK cells, has been shown to regulate the immune response during the development of *Lactobacillus casei *cell wall extract-induced coronary arteritis, an animal model of KS [18]. Many studies have attempted to identify possible genetic variants in the major histocompatibility complex region that are associated with KS and development of CAA in various populations, with conflicting results [19,20]. Recently, a functional single nucleotide polymorphism in the inositol 1,4,5-trisphosphate 3-kinase C gene, a negative regulator of T cell activation, has been linked to KS susceptibility in both Japanese and American children [21]. Genetic variations in the pathway of transforming growth factor-β, a crucial peptide with regulatory functions in T-cell activation and cardiovascular remodeling, have been proven to influence KS susceptibility, disease outcome, and response to therapy [22].

Genome-wide association studies have been performed to identify novel loci modulating the susceptibility to KS and plausible candidate gene associations have been recognized, including apoptosis regulatory genes, genes implicated in the synthesis of transcription factors and interleukin inhibitors [23]. A recent study performed in 186 Korean patients has detected two susceptibility loci, one in the 1p31 region for uncomplicated KS and a second in the 2p13.3 region for KS complicated by CAA [24].

### Atypical and incomplete variants of the syndrome

Diagnosing and treating both "atypical" and "incomplete" forms of KS remain major dilemmas for clinicians evaluating children, especially infants, with prolonged or refractory high fever. Even after 5 years of age, the diagnosis of KS can be very hard to establish. According to the American Heart Association and the American Academy of Pediatrics, "atypical" KS is defined as a child with high fever and symptoms and signs heralding the onset of the disease that are not included in the clinical major criteria of KS, such as acute abdominal pain, pulmonary signs, or gastrointestinal signs [25]. The more common atypical signs which can be observed in KS are listed in Table 2.

**Table 2:** Atypical findings of Kawasaki syndrome

Neurologic: stiff neck secondary to aseptic meningitis, facial nerve palsy, sensorineural hearing loss, extreme irritability

Renal: sterile pyuria, proteinuria, nephritis, acute renal failure

Musculo-skeletal: joint involvement (arthralgias or arthritis), leukocytosis in synovial fluid

Pulmonary: pleural effusion, lung infiltration

Gastrointestinal: abdominal pain, diarrhea, hepatitis, obstructive jaundice, hepatic dysfunction with hypertransaminasemia, gallbladder hydrops, pancreatitis

Genital: vulvitis, meatitis, urethritis, sterile pyuria

Ophthalmologic: anterior uveitis

Dermatologic: peripheral extremity gangrene, erythema multiforme-like lesions, erythema or induration at the site of bacillus Calmette-Guérin vaccination, Raynaud's phenomenon

Conversely, the term "incomplete" KS refers to patients who lack the clinical findings of the disease to fulfill the classic criteria, but present fever, at least two of the typical clinical manifestations and CAA revealed by echocardiography [26]. It is clear that the presence of coronary damage detected by ultrasound investigations may confirm KS diagnosis in many doubtful cases.

The primary goal for pediatricians should be early recognition and treatment of both atypical and incomplete cases before coronary arteries are damaged. Among all KS patients, atypical and incomplete cases have the highest risk of developing CAA if left untreated with IVIG [27,28]. In children who are suspected to have KS, acute phase reactants, in particular the erythrocyte sedimentation rate (ESR) and C-reactive protein (CRP), can provide important clues to the diagnosis, as they are often markedly elevated at least by the fourth or fifth day of illness, in comparison with levels expected in children with acute viral illnesses [29]. Laboratory parameters individually have insufficient sensitivity or specificity for a prompt diagnosis; thus, only the combination of medical history, physical examination and laboratory tests, including a high white blood cell count, ESR and CRP, could help in ruling out the other illnesses. (See Table 3 for a differential diagnosis of KS) [30]. Systemic onset-juvenile idiopathic arthritis may also resemble KS both in clinical manifestations and laboratory abnormalities [31]. Notably, a study of patients referred for the evaluation of a possible KS showed that over 40% of children with an alternative diagnosis simultaneously fulfilled the diagnostic criteria of KS [32]. Hence, atypical and incomplete KS raise criticism about the validity and suitability of diagnosis based on the classic clinical criteria. These patients with incomplete forms of the disease present not only the coronary artery involvement, but these children may also clinically display noncoronary cardiac lesions, such as pancarditis, conduction system abnormalities, and subclinical ventricular dysfunction or subtle ventricular dilations, all involvements possibly independent of CAA [33].

**Table 3:** Disorders to differentiate from Kawasaki syndrome

Viral infections (e.g. measles, infection by Adenovirus, Enterovirus or Epstein-Barr virus)

Scarlet fever

Staphylococcal scalded skin syndrome

Toxic shock syndrome

Bacterial cervical lymphadenitis

Drug hypersensitivity reactions

Stevens-Johnson syndrome

Systemic-onset juvenile idiopathic arthritis

Rocky Mountain spotted fever (*Rickettsia rickettsii *infection)

Leptospirosis

Mercury hypersensitivity reaction

Another suggestive symptom in recognizing KS is the extreme irritability of the majority of pediatric patients, which is mainly related to aseptic meningitis and is uncommon in other febrile diseases [34]. An interesting finding, due to the crossreactivity of T-cells between epitopes of mycobacterial and human heat shock proteins, is the development of erythema and induration at sites of bacillus Calmette-Guérin immunization: in countries where anti-tubercular vaccination is common this sign might aid the clinician in the diagnosis of KS [35].

### Therapy of primary Kawasaki syndrome

Since no criteria have been developed that can reliably identify children at higher risk for severe disease at the time of initial presentation, all children diagnosed with KS must be treated at the time of diagnosis. Since both KS etiology and pathogenesis are not completely clarified, the general aims of treatment are to rapidly diminish the inflammation within the vascular system and specifically in the coronary arteries, minimize the incidence and progression of CAA, and prevent arterial thrombosis by inhibiting platelet aggregation [36]. The current treatment strategy is based on IVIG therapy (2 g/kg of body weight in a 10-12 hour-infusion) and aspirin (50-100 mg/kg daily, divided in four doses), both given within the tenth day of fever onset [37]. This treatment has an overall systemic antinflammatory effect in approximately 80% of patients and reduces the aneurysm formation rate to less than 5%.

IVIG and aspirin therapy should be instituted as soon as possible when KS is diagnosed. If CAA are detected in a child prior to the child's fulfilling all KS diagnostic criteria, treatment with IVIG and aspirin should also be initiated then. When the KS diagnosis is delayed but inflammatory parameters are still high, it is however recommended to treat patients aggressively with IVIG and aspirin, especially young infants, even though the efficacy of this early treatment in preventing coronary artery injuries is uncertain [38]. Approximately 10 to 20% of children fail to respond to IVIG treatment. Several variables seem to predict this unresponsiveness, i.e. low sodium and albumin, neutrophil leukocytosis, low platelet count, high transaminases and CRP level, the day of illness at initial treatment, and patient's age [39,40]. Various studies have shown that the incidence of CAA is inversely related to IVIG dose and independent of the salicylate dose [41]. After the acute phase of KS, the aspirin dose can be reduced to 3-5 mg/kg once daily to act as an inhibitor of platelet function and this is continued for at least 6-8 weeks or longer if echocardiography shows CAA [42]. In children with either an allergy to aspirin or concomitant varicella and influenza (who may be at risk to develop Reye syndrome), clopidrogel (given at a dose of 1 mg/kg per day up to a maximum dose of 75 mg/day) might be a potential substitute [43].

Although corticosteroids are the treatment of choice in most vasculitides of adulthood, their role as a first-choice treatment for KS is controversial. A multicenter prospective randomized trial of corticosteroids in primary KS showed that the combination of IVIG and corticosteroids improves clinical course and coronary artery outcome, without causing side effects [44]. However, data from Newburger do not show a clear usefulness of adding a single dose of intravenous methylprednisolone to the conventional IVIG therapy for the routine primary treatment [45]. A recent study has detected a high regression rate of CAA, including giant aneurysms, after IVIG infusion followed by pulse intravenous methylprednisolone at the dose of 30 mg/kg for three consecutive days [46].

### Management of the refractory Kawasaki syndrome

No specific guidelines are available for the management of refractory KS patients in whom inflammatory parameters do not subside and fever persists or recurs 24 to 48 hours after IVIG infusion, with the possible increased risk of coronary damage. Most of these patients will respond to a second infusion of IVIG (at the usual dose of 2 g/kg), but up to one third of non-responders remain febrile with a persistently high serum CRP level [47,48]. Because no controlled trials have been performed, there are different therapeutic approaches in the treatment of refractory KS with thus far contradictory results. Daily high dose-methylprednisolone for a period of 1-3 days is often used in patients with a recurrent disease, as a third dose of IVIG could be both expensive and unsuccessful, but the exact benefit of methylprednisolone in the initial treatment of the disease and treatment of the refractory cases remains uncertain [49,50]. A combination of an intravenous bolus of methylprednisolone followed by oral corticosteroids progressively tapered until CRP normalization has also been administered successfully in a small group of refractory patients [51].

Infliximab, a chimeric mouse-human monoclonal antibody targeting soluble and membrane-bound TNF-alpha, has been successfully used in patients refractory to IVIG and corticosteroids, even with severe coronary artery involvement, without substantial side effects [52,53]. Nonetheless, Hirono et al. have highlighted that this drug reduces the cytokine-mediated inflammation, but does not suppress vascular cellular infiltration [54]. Another TNF-blocker, etanercept, a fusion protein combining the TNF receptor-2 with the Fc component of human immunoglobulin G1, has been administered immediately after IVIG infusion and then weekly in 17 patients aged 6 months-5 years (0.4 mg kg/dose in the first 5 patients, 0.8 mg kg/dose in the other 12). This etanercept therapy has led to a stable defervescence and no increase in coronary artery diameter or new coronary artery dilation [55]. Until future clinical trials will establish the best clinical option, infliximab and etanercept can be considered as potential alternative drugs for refractory KS. Other strategies that have proven successful in accelerating the resolution of fever in selected cases of aggressive or resistant disease include cyclosporine, low-dose oral methotrexate and plasma exchange [56-58]. Patients with severe coronary artery involvement, mainly those with giant aneurysms, require long-term anticoagulation therapy to prevent intracoronary thrombus formation, myocardial ischemia and potential risk of sudden death. Current recommendations for systemic anticoagulation include the administration of the combination of aspirin and warfarin that appear to significantly reduce the incidence of myocardial infarction [59]. Due to problems associated with warfarin use in children, such as difficulties in predicting dose response and safety concerns, other authors have shown that some advantage might derive from using low molecular weight heparin, instead of warfarin, without increasing the risk of thrombosis or bleeding [60].

### Cardiac complications and long-term atherosclerotic risk

The major sequelae of KS, such as CAA leading to potential occlusion and cardiac ischemia, are dramatically decreased as a significant result of therapy with IVIG. Coronary artery damage is regarded as the hallmark of KS and is the single risk factor that contributes the most to an increase in the mortality risk of this disease in any patient [61]. Other cardiovascular complications include pericardial effusion, myocarditis, valvulitis, mainly mitral regurgitation, and aneurysms in systemic arteries [62]. Up to 25% of untreated children will develop persistent abnormalities in the coronary arteries with inflammatory cell infiltration of the arterial wall, destruction of the internal elastic lamina, necrosis of smooth muscle cells, myointimal proliferation and subsequent occurrence of dilations or aneurysms [63].

Aneurysms are usually classified as *saccular*, if axial and lateral diameters are nearly equal, or *fusiform*, if symmetric dilation with gradual proximal and distal tapering is seen. When a coronary artery is larger than normal without a segmental aneurysm, it is considered "*ectatic*". In addition, according to the last American Heart Association statement, aneurysms can be classified as "*small*" (less than 5 mm internal diameter), "*medium*" (5-8 mm internal diameter) or "*giant*" (more than 8 mm internal diameter) [64].

Abnormal endothelial function might persist for many years after the resolution of the acute phase of KS, even in patients without detectable coronary artery damage. Patients with this abnormal endothelial function might display subclinical vasculitis many years after KS [65]. Endothelial dysfunction might also be theoretically a key event in the pathogenesis of premature atherosclerosis, coronary stiffness, and hypertension in these patients [66]. Recently, Mitani et al. assessed the coronary plaque composition and coronary artery morphology of adolescents and young adults long after KS "in vivo" by virtual histology intravascular ultrasound, comparing these results with the concomitant coronary angiographic findings. Atherosclerotic lesions were characterized by a heterogeneous intimal area, distinct from a purely fibrotic area, giving a new insight into the potential role of atherogenesis in the coronary artery remodeling after KS [67].

The long-term outcome of adults who have recovered from KS without CAA is considered excellent, though longitudinal studies are required before confirming this speculation. A heart-healthy lifestyle is recommended for all patients with a history of KS, including avoidance of smoking, low fat diet, control of blood pressure and lipid panels, and regular physical activity, unless contraindicated [68,69]. Long-term cardiologic assessment and management of patients with persistent CAA may be necessary according to the stratified risks listed in Table 4. Stress tests, computed tomography angiography (also known as multi-slice computed tomography), and magnetic resonance imaging may be helpful in this long-term followup [70,71]. The indication for coronary revascularization procedures should be considered when severe occlusions are observed in the main trunk of the left coronary artery, more than one major coronary artery or the proximal segment of the descending coronary artery. Cardiac transplantation is indicated for a small number of patients with KS presenting with severe myocardial dysfunction or severe coronary arterial lesions, for whom interventional procedures are not feasible [72,73].

**Table 4:** Cardiovascular risk stratification for patients with Kawasaki syndrome

Risk level	Therapy	Physical activity	Follow-up	Invasive testing
**I** (no coronary artery changes)	None beyond first 6-8 weeks	No restrictions beyond first 6-8 weeks	Counseling at 5-year-intervals	None

**II** (transient coronary artery ectasia)	None beyond first 6-8 weeks	No restrictions beyond first 6-8 weeks	Counseling at 3-to-5-year intervals	None

**III** (one small medium coronary artery aneurysm)	Low-dose aspirin at least until aneurysm regression is documented	For patients < 11 years: no restrictions;for patients of 11-20 years: physical activity must be guided by stress test and myocardial perfusion scan; discouraged contact or high-impact sports	Annual echocardiogram + ECG; biannual stress test and myocardial perfusion scan	Angiography, if non invasive tests suggest ischemia

**IV** (≥1 large or giant coronary artery aneurysm or multiple aneurysms without obstruction)	Long term antiplatelet therapy and warfarin or low molecular weight heparin	Contact or high-impact sports should be avoided because of risk of bleeding; other physical activity recommendations must be guided by stress test and myocardial perfusion scan	Biannual echocardiogram + ECG; annual stress test and myocardial perfusion scan	Angiography at 6-12 months after the disease

**V** (coronary artery obstruction)	Long term low-dose aspirin, warfarin or low molecular weight heparin if giant aneurysms persist	Contact or high-impact sports should be avoided because of risk of bleeding; other physical activity recommendations must be guided by stress test and myocardial perfusion scan	Biannual echocardiogram + ECG; annual stress test and myocardial perfusion scan	Angiography is recommended to address the best personalized therapeutic option

### Vaccinations in children with Kawasaki syndrome

Routine vaccinations are recommended for all individuals with KS. The schedule for administration of inactivated childhood vaccines should not be interrupted. As the passively transmitted antibodies in IVIG can block the specific immune response, it may be necessary to reschedule live viral vaccines (essentially measles and varicella-containing vaccines) to at least 9 months after IVIG administration. In Japan an interval of 6 to 11 months is recommended between IVIG infusion and live vaccine administration, but there is no final agreement for IVIG-non responders who received a second or a third IVIG infusion; if the child's risk of exposure to measles or varicella is high, the child should be immunized and then reimmunized at least 11 months after administration of IVIG [74]. The administration of the inactivated vaccine for influenza in recovered KS children is prudent, especially in children still receiving aspirin. Recent studies evaluating vaccinations side effects minimize the risk that vaccines trigger KS, though a few isolated relationships have been reported for hepatitis B and rotavirus live vaccines [75-77].

## Conclusion

Although our understanding of KS has increased enormously and many facts about KS have been elucidated, numerous dilemmas remain unsolved. The first concern is the need for the discovery of the causative agent that may allow more appropriate treatment for affected children. Secondly, there is urgency to identify specific laboratory tests to shorten the gap between the appearance of the first clinical manifestations and the diagnosis, with the possibility of reducing the occurrence of coronary damage or cardiac complications in adulthood. A critical point is related to infants younger than 3-6 months who frequently display an atypical or incomplete form of the disease, often sharing signs and symptoms with other infantile febrile illnesses mimicking KS. Therefore, it is crucial to perform early transthoracic echocardiography in all patients with suspected or established KS, and introduce the appropriate therapy even in the absence of all typical findings.

Patients with giant aneurysms require aggressive anticoagulation with lifelong follow-up, including stress tests and multi-slice computed tomography or magnetic resonance imaging. The degree to which KS is a cardiovascular risk factor for progression to atherosclerosis is under debate and long term evolution and consequences of the disease are still unknown, despite many efforts of researchers worldwide. The available data suggest that all KS patients, including those without CAA, should remain connected to their medical system, even in their transition to adult medical care, with the hope that collaborative scientific research among various centers will help define the cause and eventual prevention of the syndrome and the optimal management of current KS patients.

## Abbreviations

KS: Kawasaki syndrome; CAA: coronary artery aneurysms; IVIG: intravenous immunoglobulins; TNF: tumour necrosis factor; ESR: erythrocyte sedimentation rate; CRP: C-reactive protein.

## Competing interests

The authors declare that they have no competing interests.

## Authors' contributions

FF (submitting author) primarily created and drafted the manuscript and revised it based on co-authors' suggestions; SC and DR have been involved in the critical revision of the manuscript, giving the final approval of this final version.
